# Embedding of Active Proteins and Living Cells in Redox-Sensitive Hydrogels and Nanogels through Enzymatic Cross-Linking**

**DOI:** 10.1002/anie.201206266

**Published:** 2013-02-05

**Authors:** Smriti Singh, Fuat Topuz, Kathrin Hahn, Krystyna Albrecht, Jürgen Groll

**Affiliations:** * DWI e.V. and Institute of Technical and Macromolecular Chemistry, RWTH Aachen UniversityForckenbeckstrasse 50, 52056 Aachen (Germany); Department and Chair of Functional Materials in Medicine and Dentistry, University of WürzburgPleicherwall 2, 97070 Würzburg (Germany) E-mail: juergen.groll@fmz.uni-wuerzburg.de

**Keywords:** colloids, enzyme catalysis, gels, horseradish peroxidase (HRP), redox-sensitive materials

Redox-sensitive materials have received dramatically increasing interest over the past years.^1^ Disulfide cross-linked colloidal networks are particularly appealing, as they are rapidly reduced to thiols under the reductive environment inside cells, allowing the quantitative release of the payload incorporated within the particles.^2^ Moreover, disulfide cross-linked hydrogels as three-dimensional cell-culture scaffolds can be degraded under cytocompatible mild reductive conditions without affecting the vitality of the embedded cells.^3^

Disulfide cross-linked networks may be obtained through direct polymerization of disulfide-containing monomers, by the use of disulfide-containing cross-linkers, and by the oxidative coupling of thiol-functionalized precursors (thiomers).^4^, ^5^ Only the latter allows the covalent binding of thiol-functionalized molecules (such as cysteine-terminated peptides) to the matrix directly during network formation. The gelation of thiomers solely by exposure to air is possible but is too slow for most nanoparticle preparation techniques^6^ and leads to inhomogeneous cell distribution in hydrogels. Therefore, oxidizing catalysts like the often used hydrogen peroxide are usually applied in order to decrease oxidation times. However, the strong oxidation potential of H_2_O_2_ results in the oxidation of thiols beyond disulfides,^7^, ^8^ so that the number of possible payload molecules is restricted and the formed network may not or only partially be decomposed by mild and cytocompatible reductive stimuli.

Enzymes as biological catalysts can be used to mediate hydrogel formation with the advantages of 1) cross-linking under mild conditions, 2) unique chemo-, regio-, and enantioselectivity, and 3) no need for toxic cross-linkers. A number of studies have been reported on enzyme-mediated hydrogelation.^9^–^11^ In one example inspired by the natural gelation process of fibrin, Lutolf et al. used transglutaminase to cross-link polymers to form hydrogels. They used this process to generate cell adhesion peptide functionalized hydrogels that are also degradable by matrix metalloproteases.^12^ Approaches for the enzymatic cross-linking of hydrogels especially for biomedical applications have most recently been reviewed.^13^ This overview shows that enzymatic cross-linking has so far always resulted in networks for which degradation is either possible through enzymatically catalyzed cleavage of covalent bonds or rather harsh and not cytocompatible chemical conditions like drastic pH changes.

Horseradish peroxidase (HRP) is one of the most thoroughly studied oxidation-catalyzing enzymes.^14^ In the conventional HRP cycle the enzyme reacts with H_2_O_2_ to form highly reactive intermediates that act as powerful oxidants. In the presence of thiol groups these intermediates generate active thiyl radicals that may then dimerize to form disulfides or react with thiolates to give disulfide radicals, which are then transformed into disulfides after reaction with oxygen. Hence, addition of hydrogen peroxide is usually necessary for HRP-mediated cross-linking.^15^–^17^ Under aerobic conditions, however, there is no need for H_2_O_2_ addition as H_2_O_2_ is formed during the auto-oxidation of thiol in stoichiometric ratios.^18^ It can then act as a catalyst for disulfide formation, without the need for further addition of peroxide. Because the peroxides are generated from the thiols in a strictly stoichiometric molar ratio, no further oxidation of the forming disulfide groups occurs.

We describe herein the HRP-mediated preparation of functional, redox-sensitive disulfide-cross-linked hydrogels and nanogels through the gelation of thiomers without the requirement of H_2_O_2_. Thiol-functionalized linear poly(glycidol) (*M*_n_=6100 g mol^−1^; HS-PG) was chosen as a hydrophilic and cytocompatible gel precursor.^19^ Homogeneous solutions of HS-PG in phosphate-buffered saline (PBS) (30 wt %, pH 7.4, 8.0, 8.5) did not yield hydrogels within 24 h under ambient conditions in the absence of HRP. Upon HRP addition, gel formation was observed after 4 h only at pH 8.5, suggesting the occurrence of enzyme-triggered hydrogelation (Figure 1). Hydrogel formation was confirmed in situ during the network evolution by rheological analysis using oscillatory deformation tests. A typical gelation profile is characterized by two time phases. An initial lag phase of about 2 h, during which both moduli (elastic (*G*′) and viscous (*G*′′)) remain almost unchanged, may be related to the formation of prepolymer aggregates in the reaction system. The lag phase is followed by a log phase during which a rapid increase of *G*′ is observed, suggesting the occurrence of cross-linking reactions between the prepolymer aggregates. The gel point can be assumed at the crossover point of *G*′ and *G*′′ in the case of minimally entangled networks. Assessment of the gelation kinetics reveals a marked dependence of gelation time on HRP content. The gelation time decreased with an increasing amount of HRP and reached a plateau at 110 min for the HRP/HS-PG molar ratio of 0.08. A further increase of the HRP amount did not affect the gelation time.

**Figure 1:**
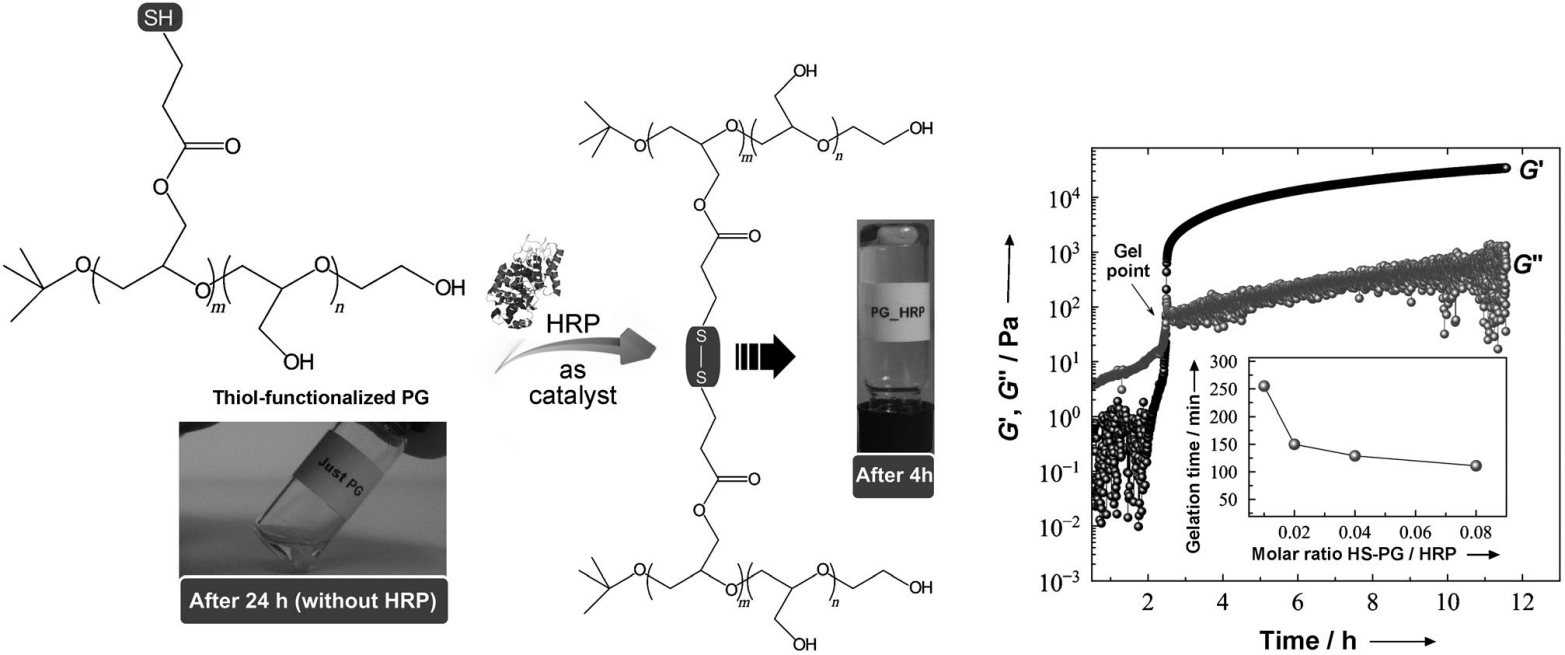
Hydrogel formation from thiol-functionalized PG upon HRP addition. The plot shows the changes in elastic *G*′, and viscous modulus *G*′′ measured at 1 Hz during the cross-linking reactions. The inset shows the dependency of the gelation time on the HRP/HS-PG molar ratio.

For comparison, an oscillatory rheological analysis with H_2_O_2_ instead of HRP as the oxidizing agent was performed (Figure S1 in the Supporting information). The gelation of HS-PG precursors in the presence of H_2_O_2_ is slower than the HRP-mediated process, but both processes yield elastic networks.

Raman spectroscopy was performed to qualitatively determine and compare the reaction products obtained by the thiomer gelation mediated with HRP and H_2_O_2_ (Figure 2). The Raman spectra of HS-PG cross-linked with H_2_O_2_ showed considerable amount of free thiol groups (2075 cm^−1^) after the stipulated time of 4 h; in comparison, in hydrogels formed by HRP cross-linking almost all of the thiol functionalities were converted into disulfides (507 cm^−1^). These results correlate well with rheological tests indicating that HRP is more effective than H_2_O_2_ as a catalyst for gel formation.

**Figure 2:**
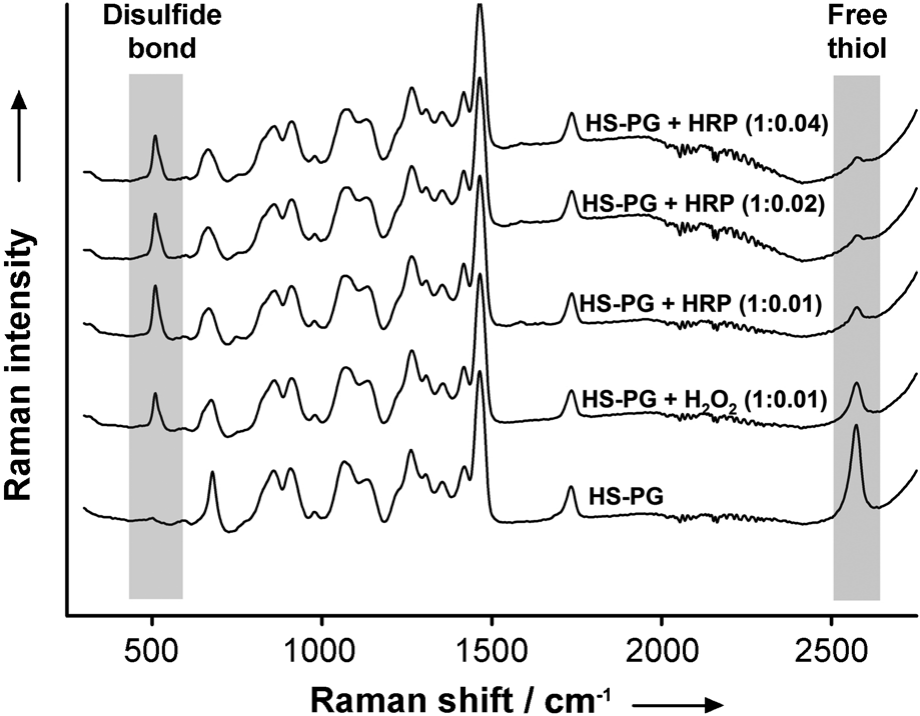
Raman spectra of HS-PG hydrogels cross-linked with H_2_O_2_ and HRP at the molar ratios indicated.

HRP-mediated thiomer oxidation was also exploited to generate hydrogel nanoparticles, so-called nanogels, by inverse miniemulsion. Cryo-scanning electron microscopy (cryo-SEM) and dynamic light scattering (DLS) techniques were applied to measure the diameter of the nanogel particles in the swollen state (Figure 3). Particle size analysis with DLS yields a z-average particle diameter of 250 nm with a polydispersity index (PDI) of 0.24. Cryo-SEM images show well-defined spherical particles with diameters *d* in the range of 200 nm<*d*<350 nm.

**Figure 3:**
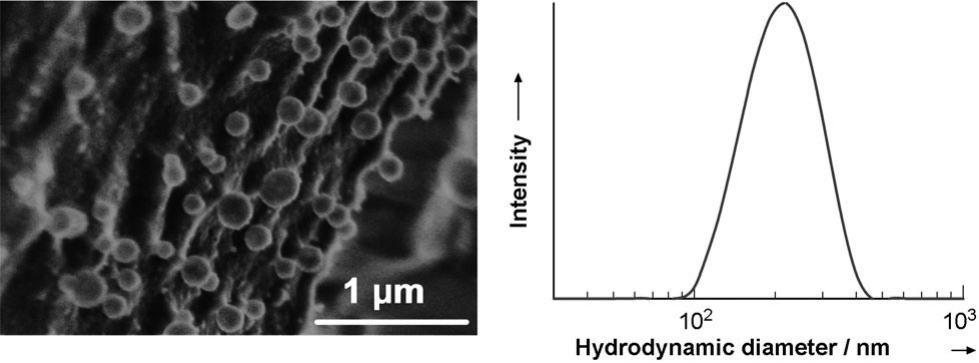
Cryo-SEM and DLS analysis of HS-PG nanogels in water.

The release of HRP from the cross-linked networks as well as the enzyme activity was analyzed by means of the HRP-mediated oxidation of colorless pyrogallol to give yellow-colored purpurogallin (see the Supporting Information). Analysis of the data revealed that in hydrogels more than 98 % of the HRP was released within 24 h (Figure 4 top). In contrast, HRP release from the nanogels was slower and incomplete, reaching a plateau at 80 % after 36 h (Figure 4 bottom). While for hydrogels roughly 45 % of the encapsulated HRP was released within 1 h, in the case of nanogels 45 % release was reached after 9 h. The fact that the nanogels were slightly brownish in color after exposure to air for 2 days (Figure S3) additionally indicates that the missing HRP (20 %) remains in the nanogels. We therefore calculated the cross-linking density of hydrogels and nanogels (see the Supporting Information). The mesh size for hydrogels was found to be 4.9 nm, while we calculated the average mesh size of HS-PG nanogels to be 3.0 nm. Since the radius of gyration of HRP is in the range of 2.5–3 nm, these calculations support the different release profiles of HRP. This may eventually be exploited for combination therapies with drugs that are activated through oxidation with HRP.^20^ HRP release from hydro- and nanogels showed in all cases catalytic activity above 90 % in comparison to the pure enzyme control (Figure 5). Enzymatic activity is thus maintained after the thiol oxidation reactions and the cross-linked network provides a suitable environment for enzyme encapsulation.

**Figure 4:**
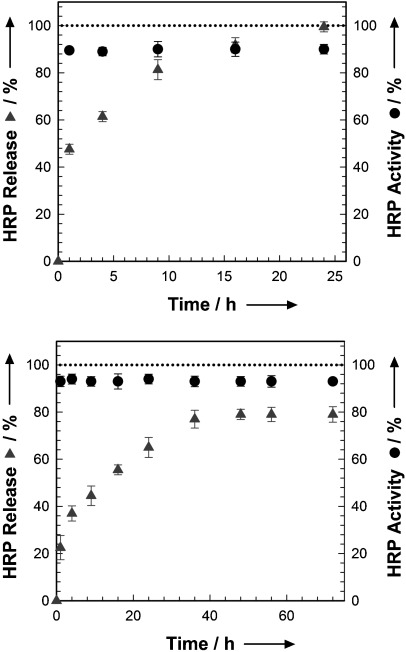
HRP release profiles and activity assays from bulk hydrogels (top) and nanogels (bottom).

**Figure 5:**
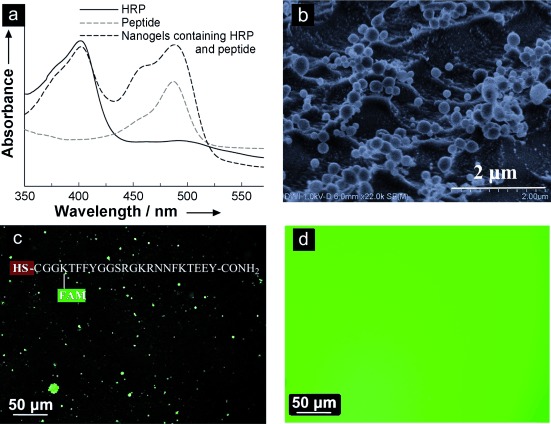
a) UV spectra of HRP, the FAM-labeled peptide sequence CGGKTFFYGGSRGKRNNFKTEEY, and peptide-conjugated HS-PG nanogels prepared by HRP-mediated oxidation; b) cryo-SEM image of peptide-labeled nanogels (diameter: 200–350 nm). c, d) Fluorescence microscopy images of the peptide- conjugated nanogels in solution before (c) and after (d) reduction with glutathione.

In order to demonstrate the cytocompatibility of the cross-linking method and the possibility of using this system for cell encapsulation, L929 cells were embedded in HRP-cross-linked hydrogels and analyzed with live/dead staining after 1 and 18 h of cell culture (Figure S4). These first experiments revealed high cell survival rates and underline the cytocompatibility of the cross-linking method and the hydrogels. The cytocompatibility of the different nanogels (1 mg mL^−1^ and 0.1 mg mL^−1^) was confirmed by cell activity assessment (WST test) using a human dermal fibroblast culture after 24 and 48 h of incubation (see the Supporting Information).

The HRP-mediated cross-linking of thiofunctional polymers can also be used to generate functionalized nanogels in a one-step procedure. We demonstrate this by adding the carboxyflourescein (FAM)-labeled model peptide sequence CGGKTFFYGGSRGKRNNFKTEEY to the nanogel preparation. This peptide bears an N-terminal cysteine that allows HRP-mediated covalent conjugation of the peptide to the nanogels. Figure 5 a shows UV spectra of native HRP, the FAM-labeled peptide, and the peptide-conjugated HS-PG nanogels after centrifugation and subsequent dialysis. Native HRP shows an intense band at 403 nm in aqueous solution (Soret band) which is characteristic for heme-containing moieties, while the peptide exhibits a maximum at 488 nm due to the presence of FAM. In the spectra of purified nanogels both bands are present, confirming enzyme encapsulation and the presence of the conjugated peptide. The labeling efficiency of the peptide estimated from a standard absorbance calibration curve measured at the 475 nm was 56 %.

The redox sensitivity of peptide-labeled nanogels was demonstrated by degradation in the presence of glutathione (GSH), a tripeptide with a free thiol group which readily cleaves disulfides to thiols and is one major component of the cytosolic redox buffer.^21^ Owing to the presence of FAM in the conjugated peptide, this process could be visualized by fluorescence microscopy. Before the degradation, particles were examined by cryo-SEM (Figure 5 b) and by fluorescence microscopy (Figure 5 c) where they appear as individual bright green spots on the dark background. This implies that the peptide is conjugated to the particles and the nonbound peptide was successfully removed by dialysis. Upon GSH addition to a final concentration of 10 mm, the fluorescent signal is spread, as shown in Figure 5 d, which depicts a homogeneous green background indicative of nanogel degradation.

In order to exploit the mild cross-linking conditions during nanogel formation, we loaded HRP-cross-linked nanogels with β-galactosidase (β-Gal), a 464 kDa homotetrameric protein that catalyzes the hydrolysis of β-galactosides into monosaccharides. This large protein is inactivated by the formation of intermolecular disulfide bonds^22^ and is thus functionally sensitive towards oxidative conditions. While encapsulation efficiencies were only 40–43 %, we could obtain nanogels in which after loading, purification, and degradation of the particles, 83–85 % of β-Gal activity was retained (Table 1; for more parameters and an extended discussion see the Supporting Information). Despite the suboptimal encapsulation efficiency, this data shows that oxidation-sensitive large proteins can be encapsulated by our method with very good preservation of protein function after release.

**Table 1:** Loading, encapsulation efficiency, and activity of β-Gal.

β-Gal inserted [mg]	Nanogel loading [mg]	Encapsulation efficiency [%]	β-Gal activity [%]
7.5	3.2	43	85
15.0	6.1	40	83

In conclusion, we have shown that HRP can be used for the mild enzymatic cross-linking of thiofunctional polymers to give hydrogels and nanogels without the need for added hydrogen peroxide. Cells can be encapsulated into hydrogels without loss of vitality, and cytocompatible and peptide-functionalized nanogels can be prepared in a one-step reaction by the addition of thiol-functionalized peptides during nanogel preparation. These nanogels show redox-sensitive degradation behavior in reductive cytosol-like environments and we have demonstrated the loading and functional release of the large model protein β-galactosidase with the nanogels using HRP cross-linking. These nanogels are thus promising candidates as controlled delivery systems.

## Supplementary material

As a service to our authors and readers, this journal provides supporting information supplied by the authors. Such materials are peer reviewed and may be re-organized for online delivery, but are not copy-edited or typeset. Technical support issues arising from supporting information (other than missing files) should be addressed to the authors.
