# Supplementary material for: Embedding of Active Proteins and Living Cells in Redox-Sensitive Hydrogels and Nanogels through Enzymatic Cross-Linking
Source: Angew Chem Int Ed Engl. 2013 Feb 5;52(10):3000–3. doi: 10.1002/anie.201206266 (PMC3601431; doi:10.1002/anie.201206266)
Supplement: Supplementary file 1 [file anie0052-3000-SD1.pdf]

Angewandte Chemie
Eine Zeitschrift der Gesellschaft Deutscher Chemiker

Supporting Information

© Wiley-VCH 2013

69451 Weinheim, Germany

Embedding of Active Proteins and Living Cells in Redox-Sensitive Hydrogels and Nanogels through Enzymatic Cross-Linking**

*Smriti Singh, Fuat Topuz, Kathrin Hahn, Krystyna Albrecht, and Jürgen Groll**

anie_201206266_sm_miscellaneous_information.pdf

Supporting Information

Materials. Linear poly(glycidol) (**PG**) (M_n = 4500 g/mol, M_w/M_n = 1.17) was synthesized *via* anionic polymerization in diglyme using potassium tert-butoxide (Aldrich) initiator according to procedure described before.[S1] N,N-dicyclohexyl-carbodiimide (DCC, Acros, 99%), 4-(dimethylamino) pyridine (DMAP, Aldrich, 99%), 3,3’-dithiodipropionic acid (DTPA, Aldrich, 99%), tris(2-carboxyethyl) phosphine (TCEP, Aldrich, 99%), dichloromethane (HPLC grade, Aldrich), cysteamine (2-mercaptoethylamine, Biochemica), 5,5’-dithio-bis(2-nitrobenzoic acid) (DTNB, Merck), anhydrous N,N-dimethylformamide (DMF, Sigma-Aldrich, 99.8%), Span 80 (Sigma), and Tween 80 (Sigma-Aldrich), pyragallol (≥ 98%, Sigma), immobilised TCEP (Thermo Scientific), AGBBB015F peptide (3230 g/mol, AplaGen), glutathione (≥ 98%, Sigma), Caffeine (Sigma) were used as received. THF was dried over $LiAlH_4$. Horseradish peroxidase with an initial enzymatic activity of 320 U/mg was purchased from CalBioChem. Dialysis membranes (MWCO: 3500 Da, 20,000 and 100,000 Da) were purchased from Spectrum Laboratories.

Dynamic light scattering (DLS). The particle sizes were measured by photon correlation spectroscopy using a Malvern Zetasizer Nano ZS at a fixed scattering angle of 173° to the incident beam. A helium/neon laser operating at 633 nm with 4.0 mW was used as a light source. For all the measurements the sample concentration of approximately 1 mg nanogel /mL water was taken. Non-invasive back scatter technology takes particle sizing to sensitivity in the 0.6 nm to 6 nm range. Disposable polystyrene cuvettes were used for measurement. ‘‘Expert System’’ software was used for data interpretation. The presented data is the average from five measurements. The DLS measurements give a z-average size (or cumulant mean)

value, which is an intensity mean and the polydispersity index (PDI). The cumulant analysis has the following form:

$$\ln(g^{(1)}(t)) = -\overline{\Gamma}t + \mu_2 t^2 + \ldots$$

where $g^{(1)}$ is the first order correlation function; is the average decay rate and first cumulant; μ_2 is the second cumulant. The value of $\mu_2 / \overline{\Gamma}^2$ is known as polydispersity index (PDI).

SEM analysis of nanogels was performed with a HITACHI S-4800 instrument in a cryo-mode at primary accelerating voltages between 1-2 kV and beam current between 1-2 A. The material is fixed on a holder and was rapidly frozen with boiling liquid nitrogen. It was then transferred to the high vacuum cryo-unit, the Balzers BF type freeze etching chamber. The cryo-chamber equipped with a knife can be handled from outside by means of a level to fracture the sample for applications in which imaging of the surface of inner structures is aimed. To remove humidity, the sample is sublimated from 5 to 15 min then the entire material is further inserted into the observation chamber. This was used to further confirm sphere size and overall morphology of the nanogels.

Synthesis of thiol functionalized poly(glycidol). Thiol functionalized linear poly(glycidol) (**HS-PG**) was prepared as described in our previous publication in a two-step synthesis.[S1] Briefly, in the first step **PG** (M_w = 4500 g/mol, DP = 60, M_w/M_n = 1.17) was cross-linked with a disulfide cross-linker 3,3'-dithiopropionic acid *via* Steglich esterification followed by reduction of the disulfide bonds to thiol groups in the second step. The reduction was carried out with aqueous solution of TCEP (1.5 eq. with respect to the disulfide units) at room temperature for 4 h under nitrogen. The pH of the solution was adjusted with triethylamine to pH = 6.5. After reduction, the solution was first dialyzed for two days at RT against aqueous HCl solution at pH ~ 3.5, followed by the dialysis in millipore water under inert gas for one day. Finally, the polymer solutions were lyophilized and stored at +4 °C under argon for

further use. The thiol content determined by ^{1}H NMR (DMSO-d6) was 25 %, (15 units) and final M_n was 6100 g/mol.

Bulk hydrogel synthesis. HS-PG (0.150 g, 3.33 × 10^{-2} mmol) was dissolved in 350 μL buffer at pH 7.4, 8.0, 8.5 and was mixed thoroughly. To this solution HRP was added in different molar ratios. The bulk gelation of **HS-PG** was induced by addition of four different equivalents of HRP with respect to thiol functionalities present in the polymer backbone as –SH : HRP: 1:0.01, 1:0.02, 1:0.04, 1:0.08. In order to test the role of HRP in gelation samples with PG was also monitored for 24 h without addition of HRP.

Rheological analysis of bulk hydrogels. Dynamic oscillatory deformation measurements were performed with a Bohlin CVR-50 (Malvern Instruments Ltd, England) in a cone-plate configuration. The upper plate (cone plate 1°, 20 mm) was set at a distance of 150 μm before the onset of the reactions. During all rheological measurements, a thin film of low-viscosity silicone oil covering the sample perimeter was used to prevent solvent evaporation. Measurements were carried out at a frequency of ω = 1 Hz and a deformation amplitude γ^{0} = 0.01 to ensure that the oscillatory deformation is within the linear viscoelastic regime.

Nanogel synthesis. Nanogels were synthesised via inverse miniemulsion method. For the preparation of the miniemulsion, surfactant (100 mg of 3:1 weight ratio of Span 80 and Tween 80) dissolved in 3.74 mL of n-hexane was used as organic phase. The aqueous phase consisted of 150 mg (3.68 × 10^{-1} mM) of **HS-PG** dissolved in 300 μL of 0.04 M PBS buffer (pH = 8.5). The organic and the aqueous phases were pre-emulsified by magnetic stirring for 10 min. After stirring the system was ultrasonicated using a Branson sonifier W450 with a ¼ ″ horn at duty cycle of 30% and output control of 90% under ice cooling. Cross-linking was initiated by subsequent addition of HRP (0.0128 g, 3.2 x 10^{-6} mmol) dissolved in 30 μL of PBS buffer (pH = 8.5) and the mixture was sonicated for another 60 s. The reaction was allowed to proceed for 50 min at room temperature with constant stirring followed by

quenching of the free thiol groups by 2-hydroxy acrylate at pH = 8. Any further oxidation was stopped by addition of 1.5 mL of acidic water (pH = 3). Separation of the nanogels was achieved by centrifugation at 10000 rpm for 30 min followed by decantation of the supernatant. Nanogels present in the aqueous layer were carefully washed with hexane (2 × 1.5 mL) and THF (4 × 2.5 mL) in order to remove the surfactants. The remaining organic solvents and acid were removed by dialysis. Purified nanogels were stored in Millipore water at 4 °C for further use.

UV-Vis spectrophotometry. UV-visible spectra were determined using a Varian Cary 100 Bio-UV-Visible split-beam spectrophotometer running with Cary WinUV scan application with a capacity of measuring 6 samples at a time. Samples were scanned at 500 nm. A high-intensity Xe flash lamp was used as the source for UV light, which permits taking 80 data per second.

Fluorescence microscopy. Zeiss Axioplan 2 upright digital imaging microscope was used for fluorescent imaging. It was equipped with a high resolution digital camera (Zeiss Axiocam HRc) and image processing software (AxioVision Release 4.7) with 100 W tungsten and mercury light sources. Axiovision software controls the stage, filter sets, and AxioCam camera provides Z-stack and multi-channel digital image capture capabilities with a resolution of 1300 × 1030 (native) up to 3900 × 3090 pixels. Microscope objectives available were 10X, 20X and 50X.

Comparison of HRP and hydrogen peroxide regarding mechanical properties of the resulting gels

As comparison to HRP cross-linked gels (see Fig. 1), an oscillatory rheological analysis with H_2O_2 instead of HRP as oxidizing agent was performed (Fig. S1). Gelation of HS-PG precursors in the presence of H_2O_2 is slower than the HRP-mediated process, but both processes yield elastic network formation.

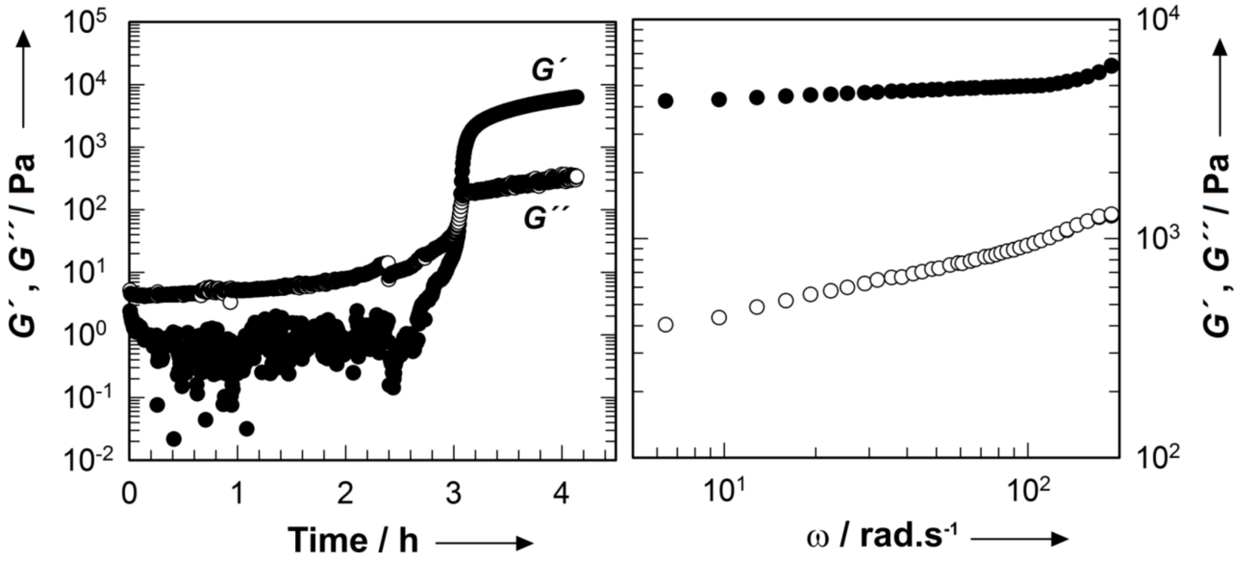

Figure S1. (Left) Elastic (G´) and viscous (G´´) modulus during gelation of HS-PG in the presence of H_2O_2. (Right) the changes in G´ and G´´ of the same sample after time sweep test as a function of frequency.

Mesh Size Calculation. The average mesh size (distance between two entanglement points) ξ for bulk hydrogels was calculated from elastic modulus *G´* based on Rubber Elasticity Theory (RET) through the following equation S1:

$$\xi = \left(\frac{G' N_A}{RT}\right)^{-1/3} \qquad \text{Eq. S1}$$

Where *G´* is the elastic modulus, N_A, R and T have their usual meanings.

There are several methods of estimating the mesh size of a nanogel; however, the Flory-Rehner model was selected due to the difficulty of measuring such parameters as the relaxed volume for different models (i.e., for the Peppas–Merrill or Brannon–Peppas equation). Thus, these values are rough estimates of the actual values.

Following the calculation of the ratio of swollen mass to dry mass, q, the polymer volume fraction in the swollen state, $v_{2,s}$ was calculated with equation S2:

$$v_{2,s} = \frac{m\bar{v}}{m\bar{v} + mq/\rho_w} \qquad \text{Eq. S2}$$

Where m is the mass of dry nanogel particles, $\bar{v}$ is the specific volume of the polymer (0.785 g/cm^3), and ρ_w is the density of water. The average molecular weight between cross-links was calculated through the following equation S3:

$$\frac{1}{\bar{M}_c} = \frac{2}{\bar{M}_n} - \frac{\left[ln\left(1-v_{2,s}\right)+v_{2,s}+\chi_1 v_{2,s}^2\right]}{\left(\frac{V_1}{\bar{v}}\right)\left[v_{2,s}^{1/3} - \frac{v_{2,s}}{2}\right]} \qquad \text{Eq. S3}$$

Where V_1 is the molar volume of the swelling agent (for water; 18 cm^3/mol), χ_1 is the Flory polymer-solvent interaction parameter (0.25 for PG) and $\bar{M}_n$ is the molecular weight number average of the polymer chains before cross-linking. Once $\bar{M}_c$ was known, the mesh size can be calculated from equation S4:

$$\xi = v_{2,s}^{-1/3}\left(\frac{C_n\bar{M}_c}{M_r}\right)^{1/2} l \qquad \text{Eq. S4}$$

Where C_n is the Flory characteristics ratio (14.6 for vinyl polymers), M_r is the average molecular weight of the repeating units, and l is the length of the bond along the polymer backbone (0.154 nm for vinyl polymers).

HRP activity assay. The enzyme activity assays of the released HRP from the cross-linked networks were performed by means of the well-established procedure of HRP mediated pyrogallol oxidation to purpurogallin.[S2] The purpurogallin which is yellow in color can be readily detected spectrophotometrically at 420 nm. One unit of peroxidase is defined as the amount of enzyme required to catalyse the production of 1 mg of purpurogallin from pyrogallol in 20 seconds at 20°C under the assay conditions described.

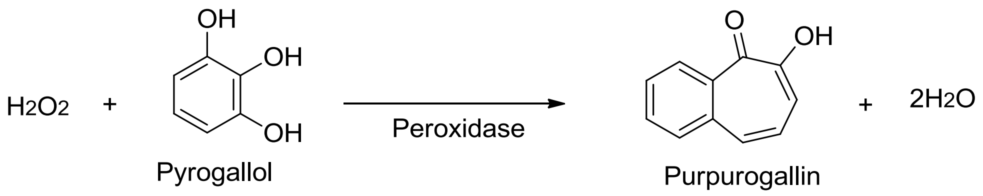

Prior to measurements the release of the HRP from hydro- and nanogels was performed as followed. Hydrogel samples were immersed at 25 °C in 10 mL of PBS buffer at pH = 6. Subsequently after 1, 4, 8, 12, 24 h the buffer solution was carefully replaced by same amount of fresh solution. After repeated washing steps nanogel particles were re-suspended in 10 mL PBS buffer at pH = 6 at 25 °C. At an interval of 1, 4, 8, 12, 24, 36, 48, 52 and 72 h nanogels were centrifuged and then again were re-suspended in fresh buffer solution. The extracted amount of HRP during the washing was subtracted from the final value. The release was monitored spectrophotometrically for all the supernatants obtained from hydro- and nanogel samples. Since the activity of enzyme is proportional to the concentration of active enzyme, this method was also used to determine the amount of active enzyme which was not released from the hydrogel and nanogels. For measurements, in 3.0 mL of reaction mixture for each blank and sample, the final concentration of 14 mM PBS buffer, 0.027 wt% hydrogen peroxide and 0.5 wt% pyrogallol was present. To achieve a temperature control both mixture in cuvettes were incubated in spectrophotometer at 20 °C for 4 min. Then 100 μL of PBS and 100 μL diluted HRP were added respectively. The change in the absorbance was continuously recorded against the corresponding control containing all of the reagents, except peroxidase, at 20 °C. The initial velocity was recorded by the absorbance-time curve. The concentration of HRP was kept so dilute that a ΔA/min was in the range of 0.02 to 0.04. The measurements were started immediately and colour densities of product solution were measured at 420 nm for 3-4 min. The values obtained in the blank reactions performed in the absence of enzyme were subtracted from all the readings. Figure S2 shows typical ΔA/min for the assay of HRP released from **HS-PG**-nanogels.

a.)   b.)

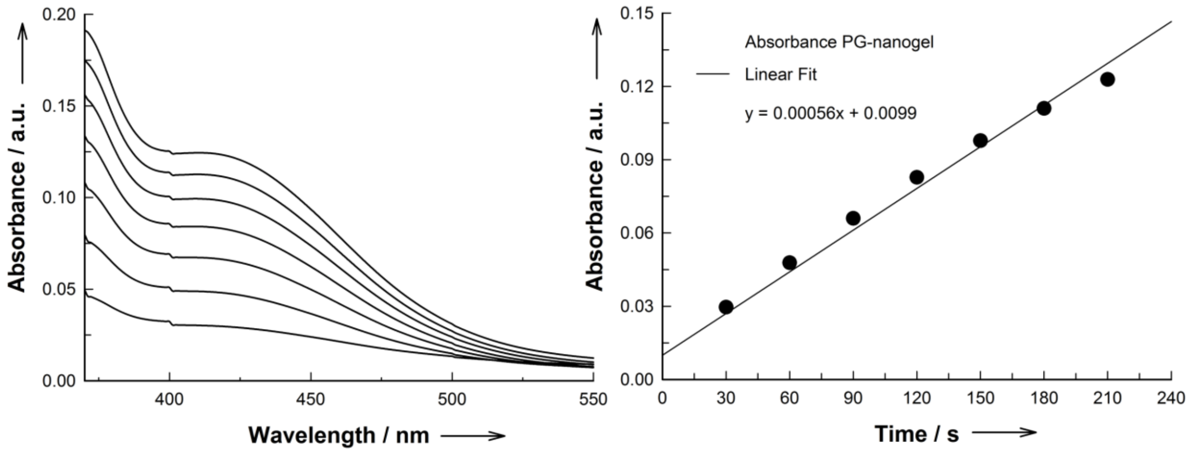

Figure S2. a) Typical UV-Vis spectra of HRP released from **HS-PG**-nanogels (release time was 24 h). The spectra from bottom to top were obtained at 30 s intervals (b) linear fit of the absorbance recorded at 420 nm

Peptide conjugation to HS-PG nanogels. Carboxyfluorescein labelled, cysteine terminated CGGKTFFYGGSRGKRNNFKTEEY peptide was used as a model peptide for HRP mediated covalent attaching to **HS-PG** nanogels. Prior to conjugation the peptide was reduced using immobilised TCEP. For coupling, 1 mg of peptide (3.096×10^{-4} mmol) was dissolved in miniemulsion aqueous phase during nanogel formation keeping the rest of the parameters as described above. After nanogel purification the amount of conjugated peptide was quantified by means of UV-Vis method. The absorption spectra were recorded between 300-600 nm with λ_{max} at 475 nm by making a standard curve for increasing peptide concentration. Total amount of the conjugated peptide was determined by measuring the absorbance of the non-conjugated peptide present in the supernatant and subtracting this value from the total amount used for coupling.

Nanogel degradation. Peptide labelled nanogels (100 mg) were degraded in the presence of water soluble reducing agent GSH (10 mM) in PBS buffer (10 mL) pH = 7.4 at 37 °C

imitating the biological conditions. Optical fluorescence microscopy was used for imaging of peptide conjugated particles before and after reduction. Images were recorded at 20-fold magnification and 50 ms exposure time (Figure S3).

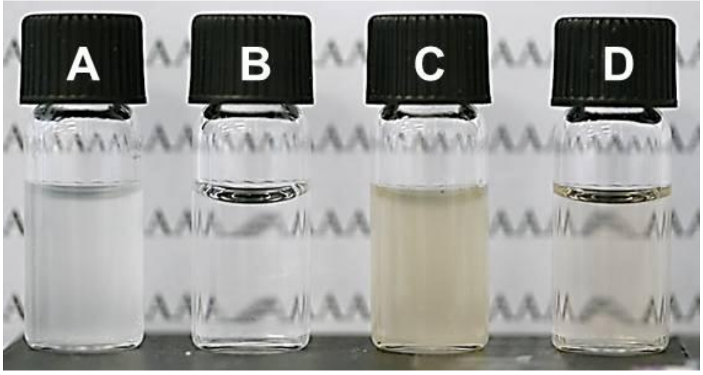

Figure S3. Nanogel suspensions before (A, C) and after (B, D) reduction with GSH, prepared by cross-linking with H_2O_2 (A, B) or HRP (C, D). The picture of particles prepared by the HRP mediated cross-linking was taken after 72 h HRP release studies. Slightly brownish color of these particles indicates HRP remaining in the particles. In contrast nanogels prepared by the cross-linking with H_2O_2 show white-milky coloration.

Cell Encapsulation in the hydrogel

Cell Encapsulation in the hydrogel was done in a double-layer approach. First SH-PG (0.025 g, 5.55×10^{-3} mmol) was dissolved in 70 µL buffer at pH 8.5. To this solution HRP (0.0003 g, 7.5×10^{-7} mmol) was added. The mixture was mixed thoroughly and was poured into the well plate. After 1h of incubation of the hydrogel, SH-PG (0.015 g, 3.34×10^{-3} mmol) dissolved in 40 µL buffer at pH 7.4 mixed with HRP (0.00018 g, 4.5×10^{-7} mmol) and 300,000 L929 mouse fibroblasts (ATCC CCL-1) were added on top of the pre-gelling hydrogel. Complete gelation of hydrogel with encapsulated cells took place within 1h. This two-layer approach ensured that no cells were in contact with the bottom of the well plate. Afterwards the gel was swollen in 1 mL DMEM plus 10 % FCS, 1 % penicillin/streptomycin, 1 % HEPES buffer,

(Invitrogen, Karlsruhe, Germany) and live/dead viability staining (LIVE/DEAD staining kit, Invitrogen, Karlsruhe, Germany) of the cells was performed according to manufacturer's protocol after 1h and 18h.

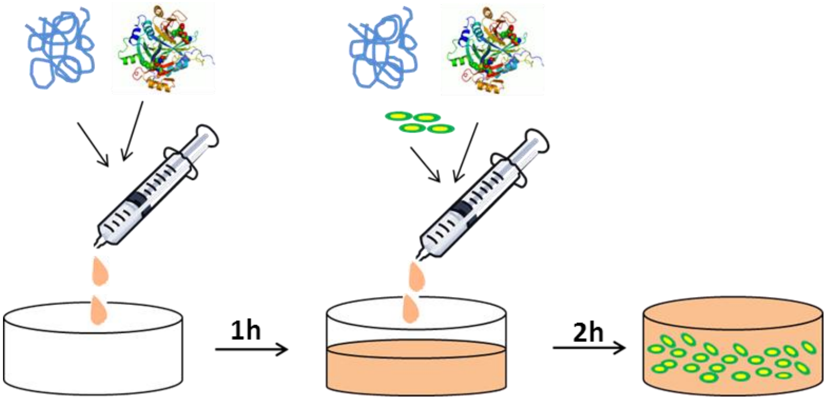

Scheme S1: Workflow of L929 cell encapsulation into the gels

Short term cell culture of up to 18 h showed very good cell viability as demonstrated by live/dead staining where green fluorescence indicate vital cells whereas red fluorescence shows permeability of the cell membrane and indicates dead cells (Figure 2). Green fluorescence of the cells underlines membrane integrity and shows vitality of the vast majority of cells (>98% after 1 h and >90% after 18 h).

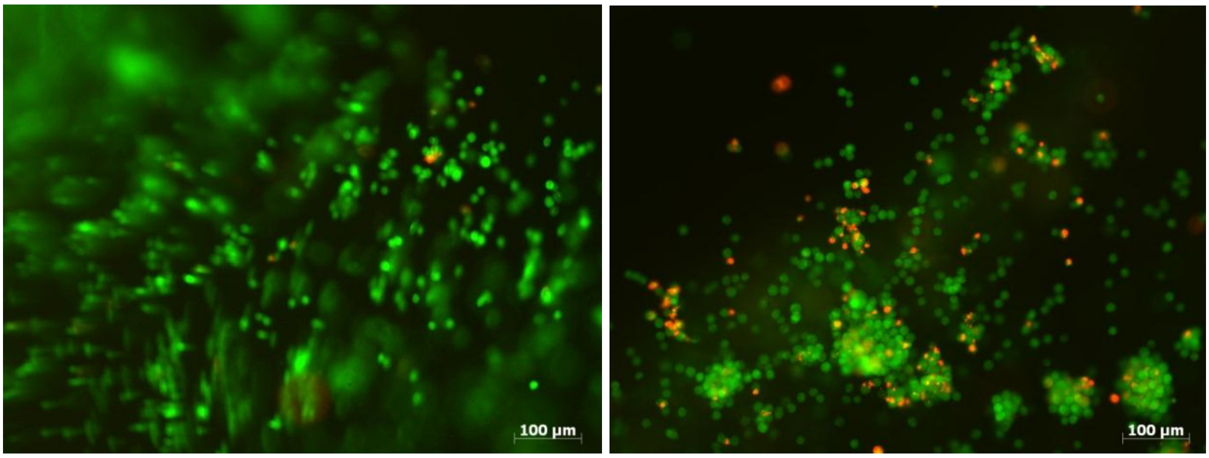

Figure S4: Typical fluorescence microscopy images of live/dead stained L929 cells after 1 h (left) 18 h (right).

Cytocompatibility assessment of the nanogels.

Experimental. Human fibroblast cell line 84-9 derived from a haemophilia patient was seeded in 48 well tissue culture plates (25000 cells per well in 500 µL DMEM with 10 % FCS, 1 % penicillin/streptomycin, 1 % HEPES buffer, all from Invitrogen Life Technologies, Karlsruhe, Germany). The cells were incubated for 24 h at 37 °C in a 5 % CO_2 humidified atmosphere. Three different nanogel samples were tested for their cytocompatibility: Nanogels where polymerization was solely mediated by HRP as prepared (HRPap), nanogels pprepared with HRP catalysis followed by dialysis against distilled water (HRPdia) as well as nanogels where cross-linking was catalysed with H_2O_2 as prepared (H_2O_2ap). All nanogel samples were dialysed against DMEM without additives for 24 h. After that FCS, pen/strep, and HEPES were added in the adequate amount and the samples were diluted 1:10 and 1:100 to reach solutions with 1 mg/mL and 0.1 mg/mL of nanogel. In addition, cytocompatibility of plain HRP (0.85 mg/mL solution diluted 1:10 and 1:100 respectively, HRPsol) was determined. The culture medium was aspirated from the cells and 500 µL of the dialysed nanogel and the HRP solutions respectively were added per well and incubated for 24 and 48 h at 37 °C in a 5 % CO_2 humidified atmosphere. Cells cultured in DMEM without any nanogel or HRP served as control.

Cytocompatibility of nanogels with 1 mg/mL and 0.1 mg/mL concentration was evaluated by means of LIVE/DEAD staining kit (L3224, Invitrogen Life Technologies, Karlsruhe, Germany), determination of cell activity using the WST reagent (Roche Diagnostics, Mannheim, Germany) according to DIN EN ISO 10993-5 as well as cell counting. For LIVE/DEAD staining, cells were washed two times with PBS and incubated with 200 µL per well of the staining solution (2 µmol calcein and 1 µmol ethidium-homodimer in PBS) for 30 min. Afterwards, the cells were analyzed by means of fluorescence microscopy (Axioimager M1, Zeiss, Germany). For WST cell activity test, the cells were incubated with

the WST reagent 1:10 in supplemented DMEM for 30 min at 37 °C. The absorption of the supernatant was quantified in a Tecan spectra fluor plus photometer (Tecan, Crailsheim, Germany) at a wavelength of 450 nm. Cell counting was performed using a CASY cell counter (Roche, Diagnostics, Mannheim, Germany). Samples for cell activity and cell counting were measured in quadruplicate. The average and standard deviation were calculated using Microsoft Excel.

Results. Nanogels prepared by HRP catalysis (HRPas, HRPdia), nanogels prepared through H_2O_2 mediated cross-linking (H2O2ap) at concentrations of 1 mg/mL and 0.1 mg/mL as well as the used HRP solutions (HRPsol) were assessed for cytocompatibility using human fibroblast cell line 84-9. After incubating the cells for 24 and 48 h, LIVE/DEAD staining showed no differences in cell viability comparing control cells to nanogel treated cells. Cell activity as assessed by the WST test was calculated in relation to cell numbers as relative absorption per cell. Cells incubated with the three different kinds of nanogels showed no significant decrease in cell activity compared to control cells after 24 h and 48 h. Addition of 1 mg/mL of dialysed nanogel (HRPdia) and nanogel with H_2O_2 (H_2O_2ap) to the cell culture medium resulted in a pronounced increase of cell activity after 24 h that lowered again after another 24 h. Cells supplemented with HRPsol showed a decrease of cell activity after 24 h. After 48 h cell activity increased again to levels similar to the ones of control cells.

Nanogels prepared with HRP and H_2O_2 did not affect cell activity and viability negatively. The results show that during the course of incubation no cytotoxic compound was released and that the nanogels proper are cytocompatible (Figure S4).

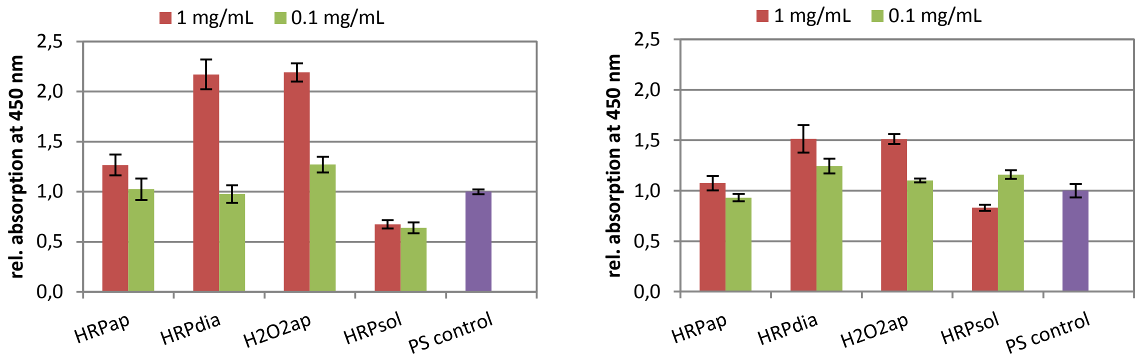

Figure S5: WST activity per cell after incubation of the different nanogel samples on human fibroblasts for 24 h (left) and 48 h (right).

β-Galactosidase loading and activity assay

Loading. For encapsulation of β-Galactosidase (β-Gal) in the nanogels, the procedure was essentially the same as the preparation of unloaded nanogels except that 5wt% and 10wt% of the enzyme with respect to the polymer weight were dissolved in the initial aqueous phase while synthesising nanogels.

Enzyme activity assay. Efficiency of enzyme encapsulation and enzyme activity were determined according to the ortho-nitrophenyl-β-D-galactopyranoside (ONPG) assay for β-Gal[1]. The enzyme β-Gal plays an important role in cellular metabolism by breaking down lactose into glucose and galactose. Enzymatic activity of β-Gal can be indirectly measured using the lactose analog ONPG which is cleaved by β-Gal to o-nitrophenyl (ONP) which has a yellow color. The absorbance of ONP can quantitatively be measured at a wavelength of 420 nm spectrophotometrically. 1 unit of β-Galactosidase is defined as the amount which hydrolyzes 1 µmol of ONPG to o-nitrophenol and D-galactose.

The assay was performed in a Z-buffer at pH 7.0. For making 500 mL Z buffer: 150 mL 0.2 M Na_2HPO_4, 100 mL 0.2 M NaH_2PO_4, 5 mL 1 M KCl, 5 mL 0.1 M $MgSO_4$ were added in

240 mL of distilled water. Just before using, β-mercaptoethanol (35 μL per 10 mL Z buffer) was added. ONPG concentration was kept at 4mg/mL while 1M Na_2CO_3 was prepared to stop the reaction.

A calibration curve for the ONPG assay was obtained for 0.5, 1.0, 1.5, 2.0, 2.5, 3.0, 3.5, 4.0, 4.5, and 5.0 mg/mL of β-Gal. 200 μL of β-Gal was added to 1000 μL of Z-buffer and incubated for 2 min at 37°C. Subsequently, 200 μL of ONPG was added and the mixture was again incubated at 37°C. The reaction was allowed to proceed for 3 min and then was stopped with the addition of 500 μL of 1M Na_2CO_3. Immediately the absorbance was measured at 420 nm. Blank measurements were made in a similar way without addition of the enzyme and values were subtracted from all the readings.

For estimation of β-Gal loading in the nanogels, supernatant from the washing of 5wt% and 10wt% of β-Gal loaded nanogel was analysed as stated above. The unloaded amount calculated from the supernatant was subtracted from the amount of β-Gal used for loading and actual loading in the nanogels was estimated. All the measurements were performed in triplicate.

For activity estimation of loaded β-Gal in the nanogel, loaded and unloaded nanogels were reduced as stated before. Reduced nanogels solution was dialysed for 12h through a 10,000 MWCO membrane. The ONPG assay was performed and absorbance was measured at 420 nm. As a blank correction, measurements made with unloaded nanogels were subtracted from the loaded ones. The activity of the enzyme was calculated according to equation S5:

$$\text{Activity} = \frac{\text{OD420} \times 1.9}{\text{t} \times \text{V} \times 0.0045} \qquad \text{Eq. S5}$$

Where 1.9 = total volume in cuvette in mL, t = time of reaction, V = volume of β-Gal used for analysis in mL, 0.0045 OD_{420}/nmol = ε_{420} ONP, path length = 1 cm; Activity = nmol / min.

Results. The bacterial enzyme β-Galactosidase (β-Gal) catalyzes the breakdown of the disaccharide lactose into the monosaccharides galactose and glucose. For an assay for β-Gal activity the synthetic substrate ortho-nitrophenyl-β-D-galactopyranoside (ONPG) with a similar structure to lactose was chosen. ONPG gets cleaved in presence of β-Gal yielding o-nitrophenol (ONP) which is yellow in the anionic form and colourless galactose. The amount of ONP formed can be measured by determining the absorbance at 420 nm and is proportional to the amount of β-Gal and the time of the reaction. The reaction is stopped by adding Na_2CO_3 which shifts the reaction mixture to pH 11. At this pH ONP is quantitatively converted to the yellow coloured anionic form and β-Gal is inactivated.

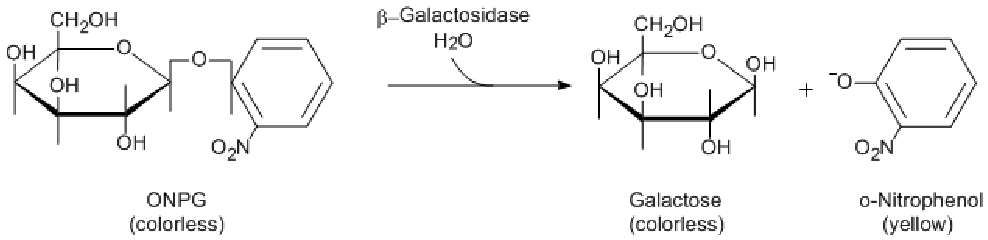

Scheme S2: Reaction of of β-galactosidase with ONPG substrate

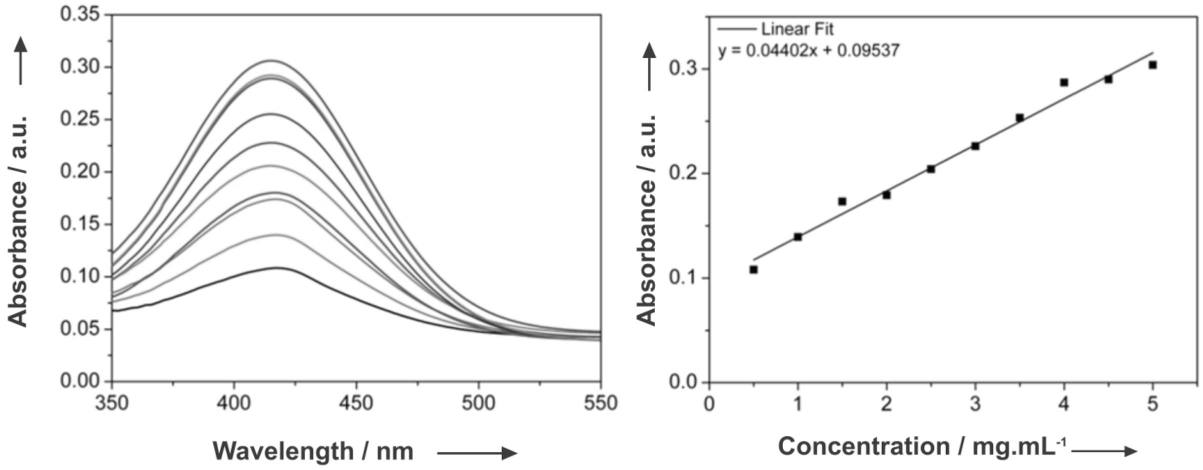

Figure S6: Typical UV-Vis spectra of ONPG assay of β-galactosidase with concentrations in range of 0.5mg/ mL to 5 mg/ mL (left panel) and linear fit of the absorbance recorded at 420 nm (right panel).

Table S1: β-galactosidase encapsulation efficiency and activity estimation

β-Gal loading [wt%]	β-Gal loaded [mg]	Loading estimated [mg]	Encapsulation efficiency [%]	% Activity	Z-Average [nm]	PDI
5	7.5	3.2	43	85	310±35	0.43±0.09
10	15.0	6.1	40	83	405±25	0.40±0.05

β-Gal activity was retained in the range of 83-85%. Prior to loading of β-Gal in nanogels, stability of only β-Gal was checked with and without sonication for 120s, the time required for preparation of nanogels via miniemulsion, and it was found that 2.5% activity of the enzyme decreased by sonication. However, there are several other factors like temperature, organic solvents etc. which may also affect protein activity during nanogel preparation.

The encapsulation efficiency of β-Gal in the nanogels was estimated to 40-43%. Low encapsulation efficiency could be attributed to the steric hindrance caused by HRP which also gets encapsulated during the nanogel synthesis.

References.

S1 Groll, J., Singh, S.; Albrecht, K.; Moeller, M. *J. Polym. Sci. A Polym.* **2009**, *20, 5543-5549.*

S2 www.sigmaaldrich.com: Enzymatic Assay of Peroxidase (EC 1.11.1.7)

S3 Miller, J. 1972. Experiments in Molecular Genetics, p. 352-355. Cold Spring Harbor Laboratory, NY
